# Atlas-based measures of left ventricular shape may improve characterization of adverse remodeling in anthracycline-exposed childhood cancer survivors: a cross-sectional imaging study

**DOI:** 10.1186/s40959-020-00069-5

**Published:** 2020-08-08

**Authors:** Hari K. Narayan, Ronghui Xu, Nickolas Forsch, Sachin Govil, David Iukuridze, Lanie Lindenfeld, Eric Adler, Sanjeet Hegde, Adriana Tremoulet, Bonnie Ky, Saro Armenian, Jeffrey Omens, Andrew D. McCulloch

**Affiliations:** 1 Department of Pediatrics, University of California San Diego, 9500 Gilman Drive #0831, La Jolla, CA 92093-0831 USA; 2 Department of Family Medicine and Public Health, University of California San Diego, 9500 Gilman Drive #0628, La Jolla, CA 92093-0628 USA; 3 Department of Mathematics, University of California San Diego, 9500 Gilman Drive #0112, La Jolla, CA 92093-0112 USA; 4 Department of Bioengineering, University of California San Diego, 9500 Gilman Drive #0412, La Jolla, CA 92093-0412 USA; 5 Department of Population Sciences, City of Hope, 1500 E. Duarte Rd, Duarte, CA 91010 USA; 6 Department of Medicine, University of California San Diego, 9500 Gilman Drive #8811, La Jolla, CA 92093-8811 USA; 7 Department of Medicine, University of Pennsylvania, 3400 Spruce Street, Philadelphia, PA 19104 USA

**Keywords:** Cardio-oncology, Anthracycline cardiotoxicity, Childhood cancer, Cardiac magnetic resonance imaging, Statistical shape atlas

## Abstract

**Background:**

Adverse cardiac remodeling is an important precursor to anthracycline-related cardiac dysfunction, however conventional remodeling indices are limited. We sought to examine the utility of statistical atlas-derived measures of ventricular shape to improve the identification of adverse anthracycline-related remodeling in childhood cancer survivors.

**Methods:**

We analyzed cardiac magnetic resonance imaging from a cross-sectional cohort of 20 childhood cancer survivors who were treated with low (< 250 mg/m^2^ [*N* = 10]) or high (≥250 mg/m^2^ [*N* = 10]) dose anthracyclines, matched 1:1 by sex and age between dose groups. We reconstructed 3D computational models of left ventricular end-diastolic shape for each subject and assessed the ability of conventional remodeling indices (volume, mass, and mass to volume ratio) vs. shape modes derived from a statistical shape atlas of an asymptomatic reference population to stratify anthracycline-related remodeling. We compared conventional parameters and five atlas-based shape modes: 1) between survivors and the reference population (*N* = 1991) using multivariable linear regression, and 2) within survivors by anthracycline dose (low versus high) using two-sided T-tests, multivariable logistic regression, and receiver operating characteristic curves.

**Results:**

Compared with the reference population, survivors had differences in conventional measures (lower volume and mass) and shape modes (corresponding to lower overall size and lower sphericity; all *p* < 0.001). Among survivors, differences in a shape mode corresponding to increased basal cavity size and altered mitral annular orientation in the high-dose group were observed (*p* = 0.039). Collectively, atlas-based shape modes in conjunction with conventional measures discriminated survivors who received low vs. high anthracycline dosage (area under the curve [AUC] 0.930, 95% confidence interval 0.816, 1.00) significantly better than conventional measures alone (AUC 0.710, 95% confidence interval 0.473, 0.947; AUC comparison *p* = 0.0498).

**Conclusions:**

Compared with a reference population, heart size is smaller in anthracycline-exposed childhood cancer survivors. Atlas-based measures of left ventricular shape may improve the detection of anthracycline dose-related remodeling differences.

## Introduction

Anthracycline chemotherapy is associated with dose-dependent cardiotoxic effects and may result in cardiac dysfunction and heart failure. This is of particular concern to the growing population of childhood cancer survivors [1]. Adverse left ventricular (LV) remodeling (changes in LV size and shape) is an important precursor to anthracycline-related cardiac dysfunction [2], and may have clinical and prognostic relevance [3, 4]. However, conventional remodeling measures, including LV mass and volume, are simplified indices that do not capture potentially important changes in LV shape. Computational modeling of 3D LV shape using cardiac magnetic resonance imaging (MRI) in conjunction with statistical atlas-based techniques for shape dimensionality reduction can assess remodeling more comprehensively than conventional measures. Importantly, this approach has resulted in the discovery of novel cardiac remodeling markers in populations comprised of individuals without cardiovascular disease and in populations with prior myocardial infarctions; however, it has not been applied to the field of cardio-oncology [5, 6]. Our objectives were to examine the utility of statistical atlas-derived measures of ventricular shape in a select cohort (*N* = 20) of anthracycline-exposed childhood cancer survivors to identify remodeling differences between 1) survivors and an asymptomatic reference population and 2) survivors exposed to low- and high-dose anthracycline therapy.

## Methods

### Study population

This is a cross-sectional analysis of cardiac MRIs in a subcohort of childhood cancer survivors derived from a parent study performed at the City of Hope Childhood Cancer Survivorship Program (Duarte, CA) and approved by its local Institutional Review Board [7]. Subjects were eligible for the parent study if they had a history of cancer diagnosed at ≤21 years of age for which they were treated with anthracyclines and were ≥ 2 years from therapy completion. For the current study, we selected 20 study participants (“survivor cohort”) who were treated with low- (< 250 mg/m^2^ [*N* = 10]) or high- (≥250 mg/m^2^ [*N* = 10]) dose anthracyclines, matched 1:1 on sex and age (+/− 0–5 years) between dose groups. Individuals who were < 30 years old were excluded to allow comparisons with the reference population, which is comprised of older adults. Individuals who were treated with concomitant chest radiation were also excluded in order to isolate anthracycline-related remodeling and avoid detection of radiation-related effects. The reference atlas was derived from a cross-sectional cohort of 1991 asymptomatic volunteers who underwent cardiac MRI (“reference population”), and was made available with permission through the Cardiac Atlas Project (www.cardiacatlas.org) [5, 8].

### Imaging acquisition and analysis

**Fig. 1:**
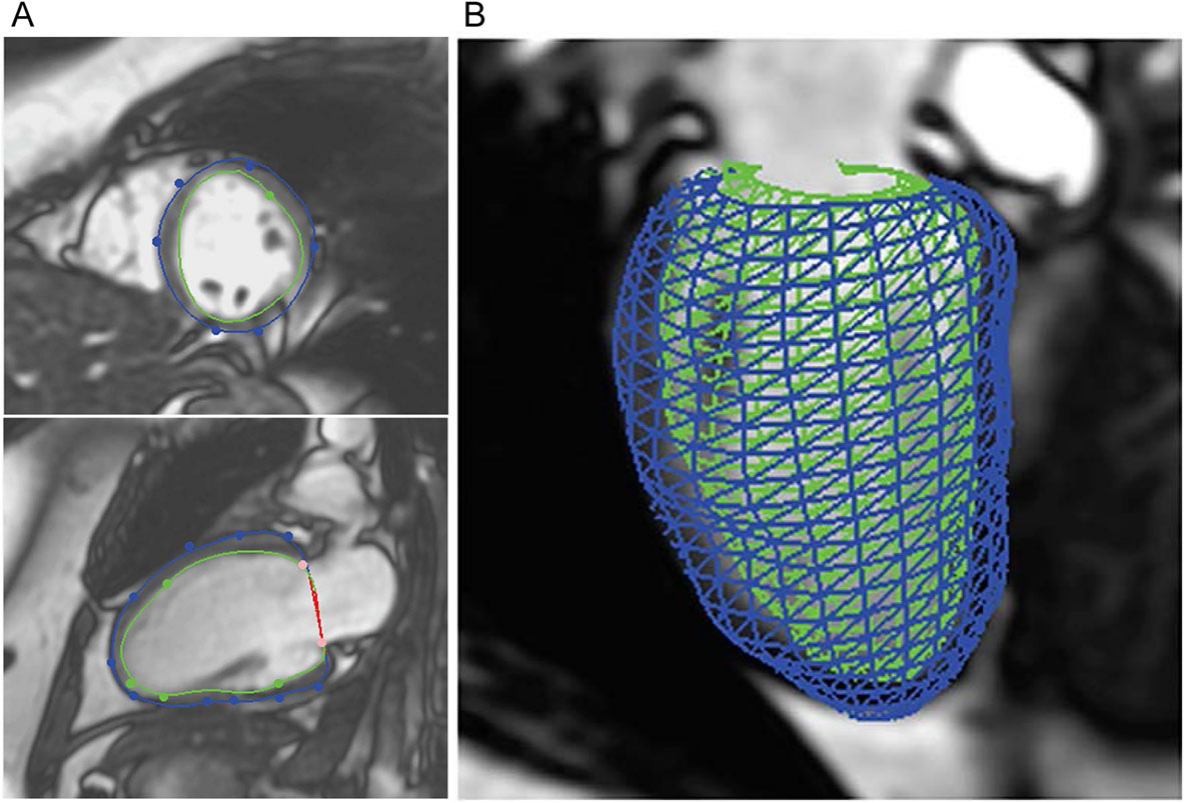
Reconstruction of Computational Models of Left Ventricular Shape. After manual identification of anatomic guide points, **a** endocardial and epicardial borders are automatically generated and manually adjusted to optimize the fit of the models to the short and long axis planes of the left ventricle, and **b** a 3D left ventricular coordinate map is generated

Each subject in the survivor cohort underwent a standard functional cardiac MRI on a 1.5 T GE Twin Speed system (GE Healthcare, Milwaukee, USA), including 2D steady state free precession cine imaging in the 2-, 3-, and 4-chamber views in addition to contiguous slices in the short axis plane spanning the base to the apex. Computational models of LV shape were reconstructed from these images using Cardiac Image Modeller software (CIM; Auckland, NZ), by a 3-step semi-automated process [5]: 1) anatomic guide points were manually identified on long and short axis images at both end-diastole and end-systole; 2) via automated endocardial and epicardial border detection, preliminary models were generated; and 3) detected borders were manually adjusted to optimize the fit of the models to each imaging plane, including adjustment for motion-related misregistration. This process results in the reconstruction of a comprehensive ~ 1700 data point 3D coordinate map of the LV (Fig. 1). Papillary muscles and trabeculations are included in the blood pool (intracavitary) in this analysis. The boundaries are constrained to fit multiple imaging planes in order to provide the optimal approximation of LV shape. As a result, they are closely aligned to imaging in each individual plane, but may have a small degree of imprecision within individual planes due to positional and heart rate differences between images. Global parameters were computed from the model, including LV end-diastolic volume (LVEDV), LV end-systolic volume (LVESV), LV mass, and LV ejection fraction (LVEF). Standard quantitative analyses of LV size and function (LV volumes, mass, and LVEF) were also performed via manually drawn endocardial and epicardial contours in Medis Suite 3.2 (Medis Medical Imaging Systems, Leiden, NL) for the purpose of validation [9]. To ensure consistency between methods, papillary muscles and trabeculations were considered intracavitary and the same end-diastolic and end-systolic frames were used in this standard post-processing analysis. A single observer (HKN) who was blinded to subject characteristics (e.g. age, anthracycline dose) performed all image analyses.

### Atlas-based left ventricular shape mode derivation

**Fig. 2:**
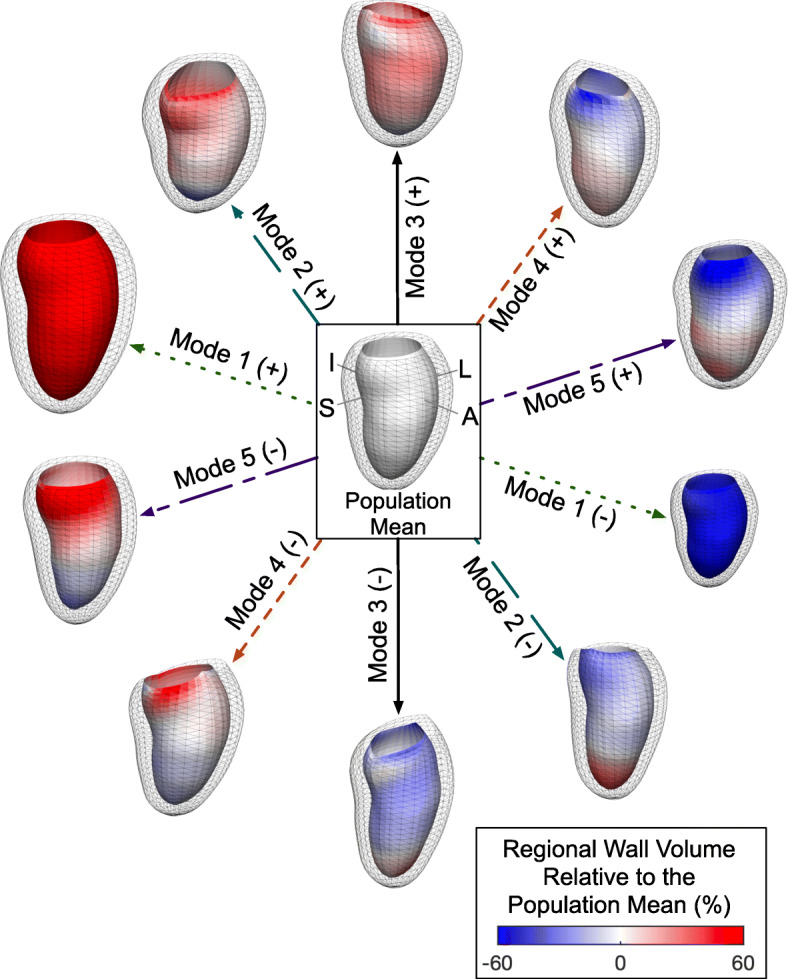
Left Ventricular End-Diastolic Shape Modes. Principal components analysis deconstructed left ventricular end-diastolic shape across the reference population into statistically independent patterns of variation (“shape modes”) [5]. The mean +/− 3 standard deviations of the five shape modes explaining the most population variation are illustrated. A blue to red color scale on the endocardium is used to depict regional wall volume relative to the population mean. The (+) indicates higher shape mode scores and (−) indicates lower values; the directionality of these values is arbitrary. Based on the qualitative appearance of the shape modes at these extremes (and as previously described [5, 11]), higher values of each shape mode correspond to findings as follows: Mode 1 to higher overall size; Mode 2 to higher sphericity; Mode 3 to higher sphericity, lower chamber length, and altered mitral annular orientation; Mode 4 to lower basal cavity diameter and altered mitral annular orientation; and Mode 5 to higher mid-cavitary size, lower mitral annular size, and lower chamber length. A: Anterior; I: Inferior; L: Lateral; S: Septal

Principal component analysis is a dimensionality reduction technique that was previously used in an unsupervised manner to deconstruct differences in LV end-diastolic shape across the reference population into a small number of statistically independent modes of variation (“shape modes”), ranked according to importance in determining population-level variation [5]. In this analysis, individual 3D LV shape models were normalized to height but retain heart size differences related to other factors [5, 10]. Each shape mode reflects the population-level variation in the data that encompasses attributes of size and both global and regional shape. After shape mode derivation, individuals are assigned a Z-score for each shape mode that indicates the number of standard deviations (SDs) his or her shape differs from population mean, allowing quantitative shape comparisons. Each mode is empirically derived and reflective of the complex relationship of a large number of data points. A visual depiction and qualitative descriptions of the first five atlas-derived end-diastolic shape modes derived from the reference population are provided in Fig. 2. By visual inspection and as previously described [5, 11], higher values of the end-diastolic shape modes correspond to LV shape features as follows: Mode 1 to higher overall size; Mode 2 to higher sphericity; Mode 3 to higher sphericity, lower chamber length, and altered mitral annular orientation; Mode 4 to lower basal cavity diameter and altered mitral annular orientation; and Mode 5 to higher mid-cavitary size, lower mitral annular size, and lower chamber length. These descriptions represent grossly observable features, and they do not capture more subtle and complex aspects of LV shape that each shape mode measures.

In our study, we used this reference atlas to enable quantitative comparisons of remodeling differences between survivors and the reference population, and among survivors exposed to low- or high-dose anthracycline therapy. We height-corrected individual survivor LV shape models and assigned individual subjects in the survivor cohort numerical Z-scores for each shape mode derived from the reference atlas, using a previously described MATLAB algorithm (MathWorks, Natick, MA) [11]. These values indicate the number of SDs that the survivor shape mode differs from the reference population mean. By definition, shape mode scores in the reference population have a mean of 0 and a SD of 1. A shape mode score of − 2 in an individual would indicate that the specific shape is 2 SDs less than the mean.

### Statistical analysis

Descriptive statistics were used to summarize the reference population and survivor cohort. To assess computational shape model reproducibility, we assess intraobserver Pearson’s correlations in a subset of five models that we generated twice. To assess computational LV shape model validity, we compared conventional measures obtained by standard analysis with those derived by the models using Pearson’s correlations.

Multivariable linear regression modeled conventional MRI measures (with LVEDV and mass indexed to height^2.7^) and the first five atlas-derived shape modes to assess the differences between the reference and survivor populations. Height-based adjustment is most accurate for left ventricular mass, which prior studies suggest is the most relevant conventional measure to anthracycline-related remodeling [3, 12, 13]. These regression models adjusted for age, ethnicity, and hypertension (past or current). To allow more meaningful examination, analyses were limited to these five shape modes, which account for a majority (76%) of the reference population variation. We performed a secondary analysis in which we compared younger age (< 50 years) and older age (≥50 years) individuals in the reference population, using descriptive statistics. We then used the same multivariable regression models described above to compare conventional parameters and atlas-based shape modes between survivors and younger individuals from the reference population.

Next, we compared the imaging parameters between survivors who received low- and high-dose anthracyclines using two-sample t-tests. To assess the collective utility of conventional and novel measures to identify anthracycline dose-related remodeling, we then used logistic regression to model the association of anthracycline exposure (low- or high-dose) with remodeling measures in three separate models: one including the three conventional remodeling measures (indexed LVEDV, indexed LV mass, and mass to volume ratio); the second including the five atlas-based shape modes; and the third including both conventional measures and the three most significant shape modes. Only the three most significant shape modes (*p* < 0.2) were retained in this third regression model to avoid overfitting. A receiver operating characteristic (ROC) curve was constructed and the area under the curve (AUC) was determined for each regression model. The AUCs for the conventional parameter model and combined model were compared using the method of Delong et al. [14]. All statistical tests were two-sided. Statistical analyses were performed using Stata 13.1 (StataCorp, College Station, Tx).

## Results

### Study population

In comparison with the reference population, childhood cancer survivors were younger (38 versus 62 years, *p* < 0.001) and were more likely to be Hispanic (70% versus 25%, *p* < 0.001), with otherwise similar characteristics (Table 1). There was a high prevalence of obesity and hypertension. Per our study design, 10 (50%) survivors had received high-dose and 10 (50%) had received low-dose anthracycline chemotherapy, with similar demographic and clinical characteristics between these subgroups apart from body mass index (BMI), which was higher in the low-dose anthracycline group. The mean age at diagnosis was 14.9 years (SD 5.7). Five (25%) survivors with hypertension were taking cardiac medications at the time of imaging; one also had a history of heart failure. One survivor was taking an angiotensin converting enzyme inhibitor, one survivor was taking an angiotensin receptor blocker, and three survivors were taking beta blockers. One additional survivor was on a study medication (carvedilol vs. placebo). Detailed demographic and clinical data for each survivor are provided in Supplemental Table 1.

**Table 1:** Subject Characteristics

Characteristic	Survivor-Reference Comparison			Survivor Cohort Compared By Cumulative Anthracycline Dose		
	Reference Population (*N* = 1991)^a^	Survivor Cohort (*N* = 20)^a^	*P*^**†**^	Low-Dose (*N* = 10)^a^	High-Dose (*N* = 10)^a^	*P*^**†**^
***Demographics***						
Age at Imaging, y	61.5 (10.2)	38.2 (6.3)	< 0.001	38.6 (6.6)	37.8 (6.3)	0.774
Sex						
Male	957 (48%)	10 (50%)	0.863	5 (50%)	5 (50%)	1.00
Female	1034 (52%)	10 (50%)		5 (50%)	5 (50%)	
Ethnicity						
White/Non- Hispanic	739 (37%)	6 (30%)	< 0.001	2 (20%)	4 (40%)	0.329
Hispanic	491 (25%)	14 (70%)		8 (80%)	6 (60%)	
Black/African American	405 (20%)	0 (0%)		0 (0%)	0 (0%)	
Chinese	356 (18%)	0 (0%)		0 (0%)	0 (0%)	
***Cancer***						
Cancer Type						
Leukemia	–	7 (35%)	–	4 (40%)	3 (30%)	0.129
Lymphoma	–	7 (35%)		5 (50%)	2 (20%)	
Sarcoma	–	6 (30%)		1 (10%)	5 (50%)	
Age at Diagnosis, y	–	14.9 (5.7)	–	13.5 (6.5)	16.4 (4.5)	0.271
Anthracycline Dose, mg/m^2^	–	255 (175, 368)	–	175 (100, 210)	368 (360, 450)	< 0.001
***Cardiac***						
Body Mass Index	27.8 (5.1)	29.1 (6.8)	0.251	32.2 (5.5)	25.9 (6.7)	0.033
Hypertension^**b**^	856 (43%)	5 (25%)	0.106	2 (20%)	3 (30%)	0.606
Heart Failure	0 (0%)	1 (5%)	0.010^**||**^	0 (0%)	1 (10%)	0.305

^a^For each group, the table depicts number (%) for categorical variables and mean (standard deviation) for continuous variables, except for anthracycline dosage, where the median (interquartile range) is shown

^b^Hypertension is defined as systolic blood pressure ≥ 140, diastolic blood pressure ≥ 90, or treatment with anti-hypertensive medication

† *P*-values for categorical variables are derived from Pearson’s chi squared tests. *P*-values for continuous variables are derived from two-sample T tests, except the *p*-value for anthracycline dose, which was derived from a Pearson’s chi-squared nonparametric equality of medians test

|| *P*-value derived from a Fisher’s exact test due to small numbers in each subgroup

### Left ventricular shape model reproducibility and validity

We first determine intraobserver reproducibility within computational shape models for conventional measures and the first five shape modes (Supplemental Table 2). Intra-observer correlations were strong for LVEDV, LV mass, LVESV, LVEF, and shape modes 1, 2, and 4; moderate for shape mode 3, and poor for shape mode 5. We then compared conventional measures derived from the computational models with those derived by standard analysis (Medis). There were strong correlations between methods for LVEF, LVEDV, LVESV, and LVEF. Overall, there was adequate reproducibility of computational models, and the models were valid in comparison to standard post-processing techniques.

### Left ventricular size and shape differences between survivors and the reference population

Conventional measures and shape modes are listed for each survivor in Supplemental Table 1. We used multivariable linear regression to model the associations of conventional measures and atlas-based shape modes with survivor cohort membership (Table 2). These regression models were adjusted for age, ethnicity, and the presence of hypertension. In comparison to the reference population, survivors had significantly lower LVEF (57% versus 63%, *p* = 0.006), indexed LVEDV (*p* < 0.001), indexed LV mass (*p* < 0.001), and a lower mass to volume ratio that approached statistical significance (*p* = 0.062). Survivors also had differences in shape mode 1 (indicating smaller overall size) and 2 (indicating lower sphericity; both *p* < 0.001).

**Table 2:** Comparison of Left Ventricular Size and Shape Between the Survivor Cohort and the Reference Population

	Reference Population (***N*** = 1991)^**a**^	Survivor Cohort (***N*** = 20)^**a**^	***P***^**†**^
*Conventional MRI Parameters*			
LVEF, %	62.9 (7.3)	56.6 (6.2)	0.006
LVEDV, ml	125.4 (31.2)	113.9 (31.1)	< 0.001
Indexed LVEDV, ml/m^2.7^	31.8 (6.5)	28.3 (7.1)	< 0.001
LV Mass, gr	126.2 (36.0)	96.5 (26.4)	< 0.001
Indexed LV Mass, gr/m^2.7^	31.8 (7.4)	23.7 (5.0)	< 0.001
Mass/Volume	1.02 (0.22)	0.86 (0.20)	0.062
*Atlas-Derived Parameters*			
Shape Mode 1	0.0 (1.0)	−0.5 (0.8)	< 0.001
Shape Mode 2	0.0 (1.0)	−1.8 (1.1)	< 0.001
Shape Mode 3	0.0 (1.0)	0.4 (1.4)	0.092
Shape Mode 4	0.0 (1.0)	0.0 (1.2)	0.412
Shape Mode 5	0.0 (1.0)	0.4 (1.1)	0.061

*LV* Left Ventricle, *LVEDV* Left Ventricular End-Diastolic Volume, *LVEF* Left Ventricular Ejection Fraction, *MRI* Magnetic Resonance Imaging

^a^For each group, the raw mean and standard deviation are shown

†Linear regression determined the association of each MRI parameter with cohort, adjusting for age, ethnicity, and hypertension, and *P*-values were derived from Wald tests

We performed a secondary analysis, restricting the reference population to those individuals < 50 years of age. We first compared characteristics of younger age (< 50 years) and older age (≥50 years) individuals in the reference population (Supplemental Table 3). Of note, the prevalence of hypertension was lower in the younger individuals (16%) in comparison with older individuals (49%). Younger individuals had slightly lower LVEF, higher indexed LVEDV, similar indexed LV mass, and higher values of shape mode 1, indicative of larger overall size. After restricting the reference population to individuals < 50 years of age, we used multivariable linear regression to model the associations of conventional measures and atlas-based shape modes with survivor cohort membership (Supplemental Table 4). We again found lower indexed LVEDV, indexed LV mass, and lower values of shape mode 1 and 2 in survivors.

### Left ventricular size and shape differences among survivors by Anthracycline dose

Next, we compared the mean values for each measure between the low- (< 250 mg/m^2^) and high- (≥250 mg/m^2^) dose anthracycline groups; there were no statistically significant differences in conventional parameters of cardiac remodeling (Table 3). However, we found significantly lower values of shape mode 4 in the high-dose group, indicative of increased basal cavity size and altered mitral annular orientation in these individuals (*p* = 0.039). To examine the collective ability of measures to identify dose-related remodeling differences, logistic regression modeled the association of anthracycline dose (low- or high-dose) with 1) conventional remodeling measures; 2) atlas-based shape modes; and 3) conventional measures and shape modes 1, 3, and 4 (the most significant shape modes in the second regression model). In ROC analysis, conventional remodeling measures did not discriminate between anthracycline dose groups (AUC 0.710, 95% confidence interval [CI] 0.473, 0.947) while the atlas-based measures had significant discriminative ability (AUC 0.880, 95% CI 0.723, 1.00). The third model, with both conventional measures and shape modes, discriminated dose exposure with an AUC of 0.930 (95% CI 0.816, 1.00), which was better than conventional measures alone (AUC comparison *p* = 0.0498).

**Table 3:** Comparison of Left Ventricular Size and Shape in the Survivor Cohort by Anthracycline Dose

	Low-Dose (***N*** = 10)^**a**^	High-Dose (***N*** = 10)^**a**^	***P***^**†**^
*Conventional MRI Parameters*			
LVEF, %	57.2 (7.3)	55.9 (5.1)	0.663
LVEDV, ml	115.3 (17.8)	112.4 (41.4)	0.840
Indexed LVEDV, ml/m^2.7^	29.5 (6.2)	27.1 (8.0)	0.459
LV Mass, gr	101.5 (19.2)	91.4 (32.4)	0.405
Indexed LV Mass, gr/m^2.7^	25.6 (3.7)	21.8 (5.7)	0.085
Mass/Volume	0.90 (0.21)	0.83 (0.18)	0.434
*Atlas-Derived Parameters*			
Shape Mode 1	−0.3 (0.6)	−0.7 (1.0)	0.311
Shape Mode 2	−1.8 (1.0)	−1.7 (1.1)	0.785
Shape Mode 3	0.7 (1.7)	0.2 (1.1)	0.419
Shape Mode 4	0.6 (1.0)	−0.5 (1.3)	0.039
Shape Mode 5	0.4 (1.2)	0.3 (1.0)	0.870

*LV* Left Ventricle, *LVEDV* Left Ventricular End-Diastolic Volume, *LVEF* Left Ventricular Ejection Fraction, *MRI* Magnetic Resonance Imaging

^a^For each group, the mean and standard deviation are shown

†*P*-values are derived from two-sample T tests

## Discussion

To our knowledge, this is the first study to utilize statistical shape atlas-derived measures to examine LV remodeling after anthracyclines. We found that 1) both conventional remodeling measures and atlas-based shape modes are significantly altered in childhood cancer survivors, demonstrating smaller size in comparison to an asymptomatic reference population; and 2) atlas-based measures improved the discrimination of remodeling differences in survivors exposed to low- versus high-dose anthracyclines in comparison to conventional measures alone. These results suggest that, with further study in larger cohorts, statistical shape atlas-based analyses may yield improved cardiac disease markers in anthracycline-exposed childhood cancer survivors.

Compared with a reference population, heart size was smaller in childhood cancer survivors by both conventional indices (mass and volume indexed to height) and atlas-based measures (shape mode 1 [overall size], also corrected for height). Although we cannot exclude the presence of unmeasured confounders in these two different cohorts, these findings are similar to prior studies and consistent with long-standing hypotheses regarding heart “shrinking” as a late effect of childhood anthracycline exposure [2, 15]. Moreover, heart size (both volume and shape mode 1) was smaller in older age individuals in our reference population, which contrasted with our finding of smaller heart size in the younger survivor cohort. The difference in shape mode 2 (lower sphericity) is also interesting and warrants further exploration in future studies. We note that in addition to sphericity, this shape mode difference corresponds to lower mass and volume and other aspects of regional shape variation, and this finding reflects more complex differences than sphericity alone.

In comparisons by dose exposure, we found that survivors treated with high-dose anthracyclines had significant differences in shape mode 4, corresponding to increased basal cavity size and altered mitral annular orientation. Relative wall thickness is thought to have particular relevance in childhood cancer survivors after anthracycline therapy [2, 16, 17], and our findings suggests that a regional increase in basal cavity diameter and a resulting reduction in basal relative wall thickness may be an important component of the adverse remodeling process. Finally, atlas-based shape modes improved the identification of anthracycline dose-related remodeling differences. These findings are consistent with prior studies, which have found that atlas-based measures are more strongly associated with health conditions where there is cardiac remodeling (e.g. post-myocardial infarction) in comparison to standard measures [5, 6]. Our findings suggest that with further research this method could yield more sensitive disease markers and pathophysiologic insight into cardiac remodeling with cardiotoxic chemotherapy.

This study has several strengths and limitations. The strengths include the application of novel techniques to comprehensively capture LV remodeling processes with demonstrated validity in comparison to standard measures. The limitations include a lack of information regarding cardiac medication usage in the reference population and subsequent clinical diagnoses in the both study groups. Owing to the relatively small sample size, there was potential for type II error; assuming a standard deviation of 1.0, we had 70% power to detect a shape mode difference of 1.17 between low- and high-dose groups. The threshold for a meaningful difference has not yet been established for these measures, but it is likely close to this effect size. Finally, there were important differences between survivors and the reference population from which the atlas-based shape modes were derived. Our finding of improved discriminative ability with atlas-based measures despite these differences provides motivation to develop new childhood cancer survivor-specific remodeling markers to better capture the most relevant aspects of shape variation.

## Conclusions

In this cross-sectional imaging study, we found that heart size was relatively small in childhood cancer survivors and that atlas-based measures improved the identification of anthracycline dose-related remodeling differences. Larger studies using these novel techniques to comprehensively assess long-term cardiac remodeling after childhood anthracycline exposure are warranted.

## Supplementary information

**Additional file 1: Supplemental Table 1.** Individual Survivor Characteristics. **Supplemental Table 2.** Reproducibility and Validity of Left Ventricular Shape Models. **Supplemental Table 3.** Reference Population Characteristics by Age Group*. **Supplemental Table 4.** Comparison of Left Ventricular Size and Shape Between the Survivor Cohort and Younger Individuals in the Reference Population.*

## Data Availability

Reference population atlas data are available through the cardiac atlas project (cardiacatlas.org). The cancer survivor cohort dataset analyzed during the current study are available from the corresponding author on reasonable request.

## Abbreviations

AUC: Area Under the Curve
CI: Confidence Interval
CV: Coefficient of Variation
LV: Left Ventricle
LVEDV: Left Ventricular End-Diastolic Volume
LVESV: Left Ventricular End-Systolic Volume
LVEF: Left Ventricular Ejection Fraction
MRI: Magnetic Resonance Imaging
ROC: Receiver Operating Characteristic
SD: Standard Deviation
