# Supplementary material for: Atlas-based measures of left ventricular shape may improve characterization of adverse remodeling in anthracycline-exposed childhood cancer survivors: a cross-sectional imaging study
Source: Cardiooncology. 2020 Aug 8;6:13. doi: 10.1186/s40959-020-00069-5 (PMC7414730; doi:10.1186/s40959-020-00069-5)
Supplement: Supplementary file 1 — Additional file 1: Supplemental Table 1. Individual Survivor Characteristics. Supplemental Table 2. Reproducibility and Validity of Left Ventricular Shape Models. Supplemental Table 3. Reference Population Characteristics by Age Group*. Supplemental Table 4. Comparison of Left Ventricular Size and Shape Between the Survivor Cohort and Younger Individuals in the Reference Population.* [file 40959_2020_69_MOESM1_ESM.docx]

**Supplemental Table 1. Individual Survivor Characteristics**

|  | Demographic and Clinical Characteristics | | | | | | | | | | | Conventional Measures | | | | | | Shape Modes | | | | |
| --- | --- | --- | --- | --- | --- | --- | --- | --- | --- | --- | --- | --- | --- | --- | --- | --- | --- | --- | --- | --- | --- | --- |
|  | Dose Group | Sex | Age | Ethnicity | Cancer | Age (Dx) | Dose | BMI | HTN | HF | Medications | EF | LVEDV | Indexed LVEDV | Mass | Indexed Mass | Mass/Vol | 1 | 2 | 3 | 4 | 5 |
| 1 | Low-Dose | M | 31.7 | Non-Hispanic | Lymphoma | 15.5 | 240 | 30.8 | No | No | No | 60.1 | 102.1 | 24.4 | 100.4 | 24 | 1.0 | -0.8 | -1.8 | -3.7 | -1.4 | 2.2 |
| 2 | Low-Dose | M | 32.3 | Hispanic | Leukemia | 13.7 | 175 | 35.6 | Yes | No | Beta Blocker | 67.2 | 103.3 | 21.6 | 143 | 29.9 | 1.4 | -0.6 | -3.2 | 2.2 | -2.7 | -0.4 |
| 3 | Low-Dose | M | 33.0 | Hispanic | Leukemia | 4.0 | 40 | 25.6 | No | No | No | 57.8 | 140.1 | 30.7 | 116.5 | 25.5 | 0.8 | -0.1 | -1.5 | 1.6 | -1.2 | 1.4 |
| 4 | Low-Dose | M | 47.8 | Hispanic | Lymphoma | 17.1 | 184 | 30.2 | No | No | No | 52.8 | 92.2 | 24.1 | 85.3 | 22.3 | 0.9 | -0.5 | -3.4 | 0.9 | -0.8 | 0.0 |
| 5 | Low-Dose | M | 48.5 | Non-Hispanic | Lymphoma | 20.0 | 150 | 26.6 | No | No | No | 45.8 | 101.9 | 25.3 | 78.4 | 19.4 | 0.8 | -0.9 | -2.5 | 2.2 | 0.1 | 1.2 |
| 6 | Low-Dose | F | 33.9 | Hispanic | Leukemia | 11.5 | 175 | 40.0 | No | No | No | 62.2 | 126.7 | 40.9 | 86.8 | 28.0 | 0.7 | 0.7 | -1.1 | 0.5 | -0.5 | 0.8 |
| 7 | Low-Dose | F | 35.5 | Hispanic | Sarcoma | 0.3 | 80 | 26.9 | No | No | No | 61.4 | 141.8 | 33.8 | 93.7 | 22.4 | 0.7 | 0.0 | -1.5 | 1.7 | 0.2 | -0.5 |
| 8 | Low-Dose | F | 38.5 | Hispanic | Lymphoma | 17.7 | 240 | 39.8 | No | No | No | 52.6 | 130.6 | 36.7 | 106.4 | 29.9 | 0.8 | 0.6 | -2.0 | 0.7 | 0.0 | -1.0 |
| 9 | Low-Dose | F | 39.2 | Hispanic | Lymphoma | 19.1 | 210 | 29.4 | Yes | No | Beta Blocker | 47 | 104.9 | 27.1 | 114 | 29.5 | 1.1 | -0.4 | -1.4 | 0.3 | 0.7 | -1.2 |
| 10 | Low-Dose | F | 45.9 | Hispanic | Leukemia | 16.2 | 100 | 37.5 | No | No | No | 65 | 109.7 | 30.8 | 90.9 | 25.6 | 0.8 | -0.5 | 0.1 | 0.6 | -0.3 | 1.8 |
| 11 | High-Dose | M | 31.0 | Hispanic | Leukemia | 11.4 | 270 | 20.3 | No | No | No | 53 | 98.5 | 21.1 | 91.8 | 19.7 | 0.9 | -1.6 | -1.5 | 1.3 | 0.5 | -0.3 |
| 12 | High-Dose | M | 33.1 | Non-Hispanic | Lymphoma | 10.0 | 360 | 32.2 | No | No | No | 63.5 | 80.2 | 17.8 | 94.2 | 20.9 | 1.2 | -1.8 | -1.0 | -0.3 | 0.9 | -0.3 |
| 13 | High-Dose | M | 34.0 | Hispanic | Leukemia | 19.5 | 495 | 36.9 | Yes | Yes | ACEi | 44.9 | 213.9 | 42.5 | 161.9 | 32.1 | 0.8 | 1.2 | 0.6 | -1.6 | 0.3 | -0.6 |
| 14 | High-Dose | M | 42.6 | Hispanic | Sarcoma | 13.2 | 360 | 27.8 | Yes | No | ARB | 58.9 | 103.9 | 23.8 | 100.4 | 23 | 1 | -0.8 | -2.0 | 0.4 | -0.9 | 1.8 |
| 15 | High-Dose | M | 45.3 | Hispanic | Leukemia | 16.3 | 360 | 29.2 | No | No | No | 60.8 | 148.7 | 33.6 | 118.1 | 26.7 | 0.8 | 0.3 | -1.4 | 0.6 | 1 | 0.6 |
| 16 | High-Dose | F | 30.1 | Non-Hispanic | Sarcoma | 11.1 | 450 | 18.5 | Yes | No | Beta Blocker | 52.6 | 73.6 | 18.3 | 57.7 | 14.3 | 0.8 | -1.6 | -2.0 | -1.4 | -2.0 | 0.2 |
| 17 | High-Dose | F | 35.4 | Hispanic | Sarcoma | 20.7 | 450 | 14.7* | No | No | No | 56.6 | 90.9 | 27.5 | 44 | 13.3 | 0.5 | -1.3 | -2.3 | 1.9 | 2.0 | 1.6 |
| 18 | High-Dose | F | 35.8 | Non-Hispanic | Sarcoma | 21.7 | 375 | 24.3 | No | No | No | 55 | 111.2 | 35.9 | 80.8 | 26.1 | 0.7 | 0.7 | -3.3 | 0.1 | 2.3 | 1.3 |
| 19 | High-Dose | F | 43.1 | Hispanic | Lymphoma | 20.5 | 300 | 27.1 | No | No | Study Drug | 57.7 | 114.3 | 26.1 | 86.1 | 19.7 | 0.8 | -0.7 | -3.2 | 1.1 | 0.4 | 0.5 |
| 20 | High-Dose | F | 47.6 | Non-Hispanic | Sarcoma | 19.0 | 420 | 28.3 | No | No | No | 56.3 | 88.9 | 24.6 | 78.9 | 21.8 | 0.9 | -0.9 | -1.0 | -0.4 | 0.8 | -1.3 |

ACEi: angiotensin converting enzyme inhibitor; ARB: angiotensin receptor blocker; Age: in years; Age (dx): age at cancer diagnosis, in years; BMI: body mass index, kg/m^2^; Dose: anthracycline dose; EF: LV ejection fraction, %; HTN: hypertension; Indexed LVEDV: in ml/m^2.7^; Indexed Mass: in gr/m^2.7^; LVEDV: in ml; M: male; Mass: in grams; F: female; Study Drug: carvedilol vs. placebo

*Extensive surgery for right lower extremity osteosarcoma and associated severe right lower extremity atrophy contributed to low BMI in this subject

**Supplemental Table 2. Reproducibility and Validity of Left Ventricular Shape Models**

| Measure | Intra-observer Model Reproducibility (n=5)* | Model-Derived Versus Standard Post-Processing (n=20)^†^ |
| --- | --- | --- |
| LVEF | 0.96 | 0.90 |
| LVEDV | 0.996 | 0.95 |
| LVESV | 0.99 | 0.96 |
| LV Mass | 0.99 | 0.95 |
| Shape Mode 1 | 0.99 |  |
| Shape Mode 2 | 0.87 |  |
| Shape Mode 3 | 0.49 |  |
| Shape Mode 4 | 0.96 |  |
| Shape Mode 5 | -0.04 |  |

* Pearson’s correlations for LV shape model intra-observer reproducibility via comparison of 5 models that were generated twice.

† Pearson’s correlations for LV shape model validity, comparing LV shape model-derived versus standard post-processing-derived conventional measures in 20 subjects.

**Supplemental Table 3. Reference Population Characteristics by Age Group***

| Characteristic | Reference Group <50 Years of Age (N=295) | Reference Group ≥50 Years of Age (N=1,635) | P-Value^†^ |
| --- | --- | --- | --- |
| Age, years | 47.4 (1.8) | 64.5 (8.5) | <0.001 |
| Sex |  |  |  |
| Male | 176 (49%) | 781 (48%) | 0.568 |
| Female | 180 (51%) | 854 (52%) |  |
| Race/Ethnicity |  |  |  |
| White/Non-Hispanic | 130 (36%) | 609 (37%) | 0.022 |
| Hispanic | 109 (31%) | 382 (23%) |  |
| Asian | 56 (16%) | 300 (18%) |  |
| Black/African American | 61 (17%) | 344 (21%) |  |
| Body Mass Index, kg/m^2^ | 27.9 (5.5) | 27.8 (5.0) | 0.731 |
| Hypertension^‡^ | 57 (16%) | 799 (49%) | <0.001 |
| LVEF, % | 61.8 (6.7) | 63.1 (7.4) | 0.002 |
| LVEDV, ml | 136 (30.8) | 123 (30.8) | <0.001 |
| Indexed LVEDV, ml/m2 | 33.7 (6.3) | 31.4 (6.4) | <0.001 |
| LV Mass, gr | 130 (36.7) | 126 (35.8) | 0.049 |
| Indexed LV Mass, gr/m2 | 31.9 (7.8) | 31.8 (7.3) | 0.804 |
| Mass/Volume | 0.96 (0.19) | 1.03 (0.22) | <0.001 |
| Shape Mode 1 | 0.3 (1.0) | -0.1 (1.0) | <0.001 |
| Shape Mode 2 | -0.1 (1.0) | 0.0 (1.0) | 0.008 |
| Shape Mode 3 | 0.1 (0.9) | 0.0 (1.0) | 0.07 |
| Shape Mode 4 | -0.2 (1.0) | 0.0 (1.0) | 0.001 |
| Shape Mode 5 | 0.1 (1.0) | 0.0 (1.0) | 0.255 |

* For each group, the table depicts number (%) for categorical variables and mean (standard deviation) for continuous variables.

† P-values for categorical variables are derived from Pearson’s chi squared tests. P-values for continuous variables are derived from two-sample T tests.

‡ Hypertension is defined as systolic blood pressure ≥140, diastolic blood pressure ≥90, or treatment with anti-hypertensive medication.

**Supplemental Table 4.** **Comparison of Left Ventricular Size and Shape Between the Survivor Cohort and Younger Individuals in the Reference Population.***

| Characteristic | Reference Group <50 Years of Age (N=295) | Survivors (N=20) | P-Value^†^ |
| --- | --- | --- | --- |
| Age, years | 47.4 (1.7) | 38.2 (6.3) | <0.001 |
| Sex |  |  |  |
| Male | 176 (49%) | 10 (50%) | 0.961 |
| Female | 180 (51%) | 10 (50%) |  |
| Race/Ethnicity |  |  |  |
| White/Non-Hispanic | 130 (36%) | 6 (30%) | 0.001 |
| Hispanic | 109 (31%) | 14 (70%) |  |
| Asian | 56 (16%) | 0 (0%) |  |
| Black/African American | 61 (17%) | 0 (0%) |  |
| Body Mass Index (kg/m^2^) | 27.9 (5.5) | 29.1 (6.8) | 0.335 |
| Hypertension^‡^ | 57 (16%) | 5 (25%) | 0.292 |
| LVEF, % | 61.8 (6.7) | 56.6 (6.2) | 0.007 |
| LVEDV, ml | 136 (30.8) | 114 (31.1) | <0.001 |
| Indexed LVEDV, ml/m2 | 33.7 (6.3) | 28.3 (7.1) | <0.001 |
| LV Mass, gr | 130 (36.7) | 97 (26.4) | <0.001 |
| Indexed LV Mass, gr/m2 | 31.9 (7.8) | 23.7 (5.0) | <0.001 |
| Mass/Volume | 0.96 (0.19) | 0.86 (0.20) | 0.322 |
| Shape Mode 1 | 0.3 (1.0) | -0.5 (0.8) | <0.001 |
| Shape Mode 2 | -0.1 (1.0) | -1.8 (1.1) | <0.001 |
| Shape Mode 3 | 0.1 (0.9) | 0.4 (1.4) | 0.192 |
| Shape Mode 4 | 0.2 (1.0) | -0.0 (1.2) | 0.404 |
| Shape Mode 5 | 0.1 (1.0) | 0.4 (1.1) | 0.499 |

* For each group, the table depicts number (%) for categorical variables and mean (standard deviation) for continuous variables. For all parameters (including MRI measures), the raw mean and standard deviation are shown.

† For demographic and clinical characteristics, P-values for categorical variables are derived from Pearson’s chi squared tests and P-values for continuous variables are derived from two-sample T tests. For MRI measures, linear regression determined the association of each MRI parameter with cohort, adjusting for age, ethnicity, and hypertension, and P-values were derived from Wald tests.

‡ Hypertension is defined as systolic blood pressure ≥140, diastolic blood pressure ≥90, or treatment with anti-hypertensive medication.
